# Assessing the clinical utility of the severity dependence scale for benzodiazepine use disorder

**DOI:** 10.4102/sajpsychiatry.v27i0.1571

**Published:** 2021-07-28

**Authors:** Karishma Lowton, Gaveeta Chiba

**Affiliations:** 1 Department of Psychiatry, Faculty of Health, University of the Witwatersrand, Johannesburg, South Africa

**Keywords:** benzodiazepine, benzodiazepine addiction, benzodiazepine use disorder, severity dependence scale, South Africa

## Abstract

**Background:**

Benzodiazepines are often used as a part of mental health pharmacological management; however, often when prescribed for extended periods, they increase the risk of benzodiazepine use disorder (BUD). Clinical interviews are at the centre of diagnosing this disorder. However, in addition to clinical assessment a simple, validated questionnaire conducted by any healthcare professional may aid in screening for BUD and referral for further management.

**Aim:**

To compare the accuracy of the severity dependence scale (SDS) as a screening tool for BUD against the standard clinical interviews using the Diagnostic and Statistical Manual of Mental Disorders, edition 5, (DSM 5) checklist amongst benzodiazepine users with primary psychiatric disorders.

**Setting:**

Outpatient psychiatric clinic in South Rand Hospital, Johannesburg, South Africa.

**Methods:**

A cross-sectional study was conducted, once informed consent was attained, looking at demographic and clinical profiles of benzodiazepine users. Clinical interviews were conducted in 81 patients who completed the SDS. In comparing the results of the SDS and clinical interview outcomes, chi-square tests were used to determine an association between categorical variables. A receiver-operating characteristic (ROC) curve was generated in determining the cut-off score in the SDS with the highest sensitivity and specificity.

**Results:**

This study indicated that a cutoff score of greater than or equal to six of the SDS showed 86% sensitivity and 90.3% specificity compared to a diagnosis of BUD made with clinical interview. The only categorical variables of marginal significance (*p*~0.06) in comparison to a BUD diagnosis were with benzodiazepine type (oxazepam) and longer duration of use (greater than 24 months).

**Conclusion:**

This study identified the SDS as a useful screening tool for BUD with a high sensitivity and specificity compared to interview outcomes. Statistically, correlates were identified between duration and type of benzodiazepine prescribed and BUD suggesting emphasis on these factors when prescribing benzodiazepines.

## Introduction

Benzodiazepines have been widely used for the treatment of various medical and psychiatric conditions.^1^ However, there is no definitive evidence in support of its long-term use considering their side effect profile and issues with dependence.^2,3,4,5,6^ Indications for use in psychiatry range from sedation to management of sleep and anxiety.^3,4^ However, regarding these particular indications, research outcomes remain mixed indicating that chronic benzodiazepine use may exacerbate anxiety, sleep and depressive-related symptoms.^5^

Common side effects reported may include somnolence, reduced motor dexterity, speech or visual impairments, affective dysregulation and erectile difficulty.^5^ Cognitive adverse effects range from confusion, disorientation, inattention, impaired concentration in the acute phase with research suggesting links between chronic use and an increased risk of neurocognitive disorders.^7^ This was further highlighted by Rowan et al. who proposed an increased risk in neurocognitive impairment in people living with HIV (PLWH) who use benzodiazepines.^8^

A United States of America study highlighted that chronic benzodiazepine users are estimated at 500 000 to 1 million in the United Kingdom, 4 million in the United States of America and several million worldwide, of which at least 50% of these users are dependent.^9^ In appraising international data outside the United States of America, the Netherlands-based study by Kan et al. indicated a prevalence of benzodiazepine use disorder (BUD), ranging from 40% to 97% in chronic benzodiazepine users.^10^ Using data from 23 specialist treatment centres in a Cape Town study, Myers et al. showed that benzodiazepines contributed to 46.4% (primary drug) of over the counter and prescription misuse.^11^ Although epidemiological research is limited, based on findings in the study by Myers et al., it is expected that trends in chronic users in South Africa may mimic international findings.

Failure to recognise the condition may contribute to under-reporting of BUD, impact on prescribing etiquette and may be one of the most common barriers to best practices in addressing BUD. One medium of addressing this may be the use of screening questionnaires highlighting a possible use disorder that warrants further assessment and management. A search revealed various questionnaires including the benzodiazepine dependence questionnaire that was found to be a valid and reliable tool in assessing benzodiazepine dependence.^12^ However, a 30-item questionnaire requires longer completion times and certainly poses more challenges in terms of translation considering our multi-lingual and multi-cultural population. In the climate of South African public healthcare with limited resources, time or availability for training, a shorter, easy to administer screening questionnaire would certainly be more useful.

A literature review revealed two studies utilising the severity dependence scale (SDS), a short, five-item, self-report questionnaire that displayed value in assessing dependence in patients using benzodiazepines on a chronic basis.^13,14^ De Las Cuevas et al.^13^ compared the SDS to the composite diagnostic international interview in 100 chronic benzodiazepine users at an outpatient mental health clinic in the Canary Islands. They found that the SDS, using a score greater than 6, was able to correctly identify 92% of patients who met the criteria for benzodiazepine dependence. Similar findings were displayed in a Taiwanese study by Tsai et al.^14^ who displayed an SDS cut-off point of 7 or higher showing high diagnostic utility with a sensitivity of 80.5% and a specificity of 85.7% in identifying problematic benzodiazepine (BZD) users using the mini international neuropsychiatric interview.

Although the SDS has not been validated in developing countries for BUD, it has been shown to display utility for other substances as seen in an Ethiopian study by Manzar et al.^15^ They concluded that SDS-Khat showed adequate psychometric validity for the psychological assessment related to the severity of khat addiction in polysubstance users.

Considering the importance of early identification and referral of patients with BUD, the primary aim of this research was assessing the validity of the SDS as a screening tool in benzodiazepine users at an outpatient psychiatric facility. Although this scale should not displace clinical interviews, it could be useful in aiding general practitioners and other non-psychiatry healthcare workers in the recognition of BUD. Furthermore, in South Africa, where access to psychiatric care is limited in certain areas, this scale could be useful as a measure of BUD if no psychiatrist were available to administer standardised interviews.

## Objectives

We aimed to compare results of the SDS in benzodiazepine users with a standardised interview using the *Diagnostic and Statistical Manual of Mental Disorders, edition 5*, (DSM 5) criteria in an attempt to determine utility as a screening tool. Additionally, we sought to describe any significant clinical or demographic factors associated with those who, on interview, met the criteria of BUD.

## Method

### Setting and participants

The research was a cross-sectional, descriptive study that was completed at the South Rand outpatient, adult psychiatry clinic. The clinic is located in the south of Johannesburg and services a total of 112 psychiatric patients of various demographic backgrounds. This facility primarily focuses on care of stable, psychiatric patients who do not require secondary- or tertiary-level psychiatric care. Referrals into the clinic arise from the general outpatient’s clinic and ‘step-down’ referrals from other psychiatric facilities, once patients are stable. Transfers out of the clinic include patients who may be managed at primary care level, for example, mild depression or patients who may require advanced psychiatric care beyond the scope of the treating doctor, often a registrar. For the estimation of sensitivity and specificity at 85% with 10% precision and a 95% confidence interval, given an anticipated prevalence of the BUD diagnosis of approximately 60%, a sample size of 80 was required based on sensitivity and 126 for specificity. The actual sample size of 81 used in this study corresponds to the requirement for sensitivity and corresponds to a precision of 12.5% for specificity.

### Questionnaire

The SDS consists of five questions to which the participant answered never to almost never (scored 0), sometimes (scored 1), often (scored 2) or always (scored 3). The questions included:

- Do you think your use of tranquilisers is out of control?
- Did the prospect of missing a dose make you anxious or worried?
- Did you worry about your use of tranquilisers?
- Did you wish you could stop?
- How difficult would you find it to stop or go without your tranquilisers?

Considering the local, cultural, educational, and social diversity of participants in the South African setting, the investigator encouraged and reassured participants regarding the questionnaire and terms used. Assistance with any queries regarding the research was readily addressed by the debriefed clinic staff.

Since the inception of the SDS, studies^13,14,15,16^ have utilised it in research for the dependence of various substances. These pre-DSM 5 era-based studies assessed utility of the SDS as a screening tool in assessing dependence as opposed to substance abuse. Considering changes in the *DSM 5*, the dependence and abuse criteria have been encompassed under substance use disorders (SUD) with the emphasis of the SDS remaining more on the psychological sequelae of use disorders. However, considering that it has displayed adequate test–retest reliability and validity^13,14,15,16^, it is proposed to be adequate in screening for substance use disorders as it has been for substance dependence. The cut-off scores, vary in the research on other substances, however, have remained closely replicable in benzodiazepine studies conducted by De Las Cuevas et al.^13^ and Tsai et al.^14^

### Procedure

Adult patients, on benzodiazepines for longer than 3 months, who expressed their comfort with understanding, reading and writing in English were invited to participate. Patients with a working diagnosis of intellectual disability, epilepsy or substance withdrawal were excluded as the focus of the study remained on benzodiazepine users with established primary psychiatric illnesses only. Patients were provided information before obtaining consent explaining the process of the study. This was further reiterated in a detailed, informed consent document. Capacity to participate was primarily based on a stable condition per the previous review by the treating team and the participant’s ability to display a basic understanding of the study to the investigator before consenting.

Demographic details and information regarding type, dose and duration of benzodiazepine were obtained from the prescription charts. Clinical interviews were carried out by a single psychiatry registrar with adequate training to establish the presence of BUD. The interview times approximated 10 min, and interview questions were guided by the *DSM 5* checklist to evaluate for symptoms. To avoid procedural and response bias, participants were encouraged to independently complete an English version of the SDS, results of which the interviewer was blinded. Each questionnaire and checklist were allocated study numbers for comparison of results once data collection was completed.

Data collection was completed from 05 March 2016 till 05 September 2016, once all possible consenting participants were recruited. Results of the questionnaire were tallied by the principal investigator and compared to the findings from the clinical interview that was then captured on Microsoft excel sheets.

### Data analysis

Data analysis was carried out using SAS version 9.4 for Windows. The sensitivity and specificity (together with their 95% confidence intervals) of the SDS in identifying the various diagnoses were calculated, with the diagnostic interview used as the reference standard. Receiver-operating characteristic (ROC) curves that are a plot of the true-positive rate against the false-positive rate for the different possible cutoff points of a diagnostic test were generated.

For establishing an association between BUD and study variables (age, gender, highest level of education, employment status, relationship status, diagnosis, type of benzodiazepine, duration of benzodiazepine use, dose of benzodiazepine used), we required mostly chi-square tests, predominantly with two categories × 2 categories (other study variables).

### Ethical considerations

This study was approved by the Ethics Committee of the University of the Witwatersrand (clearance number: M150924). Although patients were assured that participation was voluntary and would not immediately affect their management, they were also informed that should concerns arise regarding BUD, their relevant treating doctor would be notified so that necessary referral and management would follow.

## Results

Of the 112 files reviewed, 81 patients fulfilled the study eligibility criteria and consented to the study. It was interesting to note that in the total number of patients attending this clinic, 72.3% were prescribed benzodiazepines.

Of the 81 participants, 61.7%, following clinical interviews, received a diagnosis of BUD. Of these BUD patients, benzodiazepine type and duration of use were marginally significant (*p*~0.06), suggesting that BUD were associated more with oxazepam use and with longer benzodiazepine use. There were no significant associations between the presence or absence of BUD and age, gender, ethnicity, highest level of education (HLOE), employment status, relationship status, dose of benzodiazepine and psychiatric diagnosis The demographic characteristics of patients meeting the criteria of BUD and those who did not are compared in Table 1.

**TABLE 1:** Associations between variables and the presence or absence of benzodiazepine use disorder in the study population.

Variable	Category	BUD diagnosis				
		No (*N* = 31)		Yes (*N* = 50)		*p*-value for between-group test
		*n*	%	*n*	%	
Gender	Female	21	67.7	34	68.0	> 0.99
	Male	10	32.3	16	32.0	
Ethnicity	White	22	71.0	36	72.0	0.80
	Black	6	19.4	6	12.0	
	Coloured	2	6.5	5	10.0	
	Indian	1	3.2	3	6.0	
Highest level of education	Primary	1	3.2	5	10.0	0.56
	Secondary	24	77.4	38	76.0	
	Tertiary	6	19.4	7	14.0	
Employment status	Employed	6	19.4	7	14.0	0.55
	Unemployed	25	80.6	43	86.0	
Relationship status	Married	10	32.3	16	32.0	> 0.99
	Single	21	67.7	34	68.0	
Benzodiazepine type	Oxazepam	4	12.9	16	32.0	0.066
	Clonazepam	27	87.1	34	68.0	
Benzodiazepine dose	≤ 0.5	13	41.9	15	30.0	0.083
	1–2	14	45.2	17	34.0	
	2.5 or more	4	12.9	18	36.0	
	5–15	1	25.0	6	37.5	0.524
	20–30	2	50.0	9	56.3	
	> 30	1	25.0	1	6.20	
Benzodiazepine use duration	≤ 24 m	11	35.5	8	16.0	**0**.**060**
	> 24	20	64.5	42	84.0	
Psychiatric diagnosis	Depression	8	25.8	21	42.0	0.16
	Psychotic	15	48.4	14	28.0	0.095
	Bipolar	7	22.6	6	12.0	0.23
	Anxiety	3	9.7	9	18.0	0.35
	Trauma	0	0.0	3	6.0	0.28
	Personality	4	12.9	8	16.0	0.76
	Subs	0	0.0	3	6.0	0.28

BUD, benzodiazepine use disorder.

In comparing results of the SDS and clinical interviews, 14% of the patients who had a BUD diagnosis recorded SDS scores lower than the cut-off of 6, whilst 10% of patients who recorded SDS scores equal or higher than the cut-off of 6 did not receive a BUD diagnosis. The positive predictive value (the probability that, if someone is scored as having BUD according to the test, actually have BUD) was 93%, whilst the negative predictive value (the probability that, if someone is assessed as not being a case, the chance that indeed they are not) was 80%.

Choosing a cut-off of 6, the sensitivity (the rate of true positives) was 86%. For the same cut-off, the overall specificity (the rate of true negatives) was 90% (Table 2).

**TABLE 2:** Representation of sensitivity and specificity of scores.

SDS cut-off score	Sensitivity (%)	Specificity (%)	Findings
0	100.0	0.0	-
1	100.0	25.8	-
2	100.0	41.9	-
3	100.0	58.1	-
4	98.0	71.0	-
5	92.0	77.4	-
6	86.0	90.3	-
7	74.0	93.5	-
8	66.0	93.5	-
9	44.0	93.5	-
10	22.0	100.0	-
11	16.0	100.0	-
12	8.0	100.0	-
13	4.0	100.0	-
14	2.0	100.0	-
15	2.0	100.0	-
ROC AUC	-	-	0.932
Optimal cut-off	-	-	≥ 6
Sensitivity and specificity at cut-off (95% CI)	86.0 (73.3 – 94.0)	90.3 (74.3 – 98.0)	-

SDS, Severity dependence scale.

**FIGURE 1:**
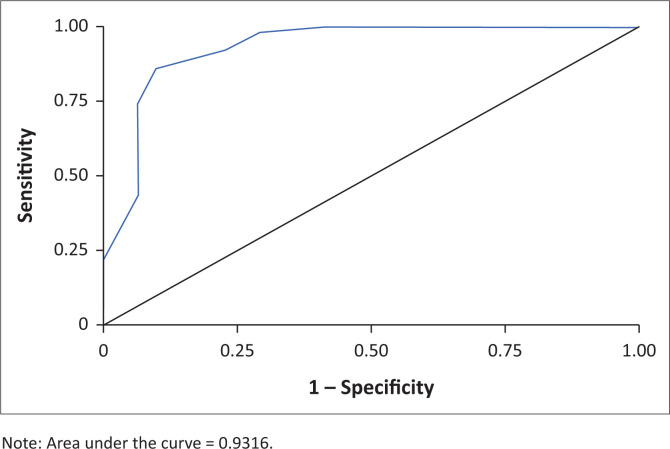
Receiver-operating characteristic curve graph.

In generating the ROC curve graph (Figure 1), the area under the curve (AUC) was calculated at 0.932, indicating that the SDS using a cut-off score of 6 can correctly discriminate patients with a diagnosis of BUD compared to using the *DSM 5* criteria in 93% of cases.

## Discussion

This study found that 61.7% of the sample studied met the criteria of BUD. Marginally significant risk factors in this population entailed the duration and type of benzodiazepine used. In recognising BUD, a cut-off point of 6 or higher on the SDS had a high diagnostic utility (AUC = 0.932), a sensitivity of 86% and a specificity of 90%.

The prevalence of BUD in this population was found comparable to studies internationally and statistics derived by Myers et al. in the South African setting^11^ with previous studies providing ranges from 40% to 97%.^9,10^ Previous research has identified BUD risk factors including older age, females, prescription of multiple BZDs and bigger doses.^14^ These were not found in this study, and variable analysis may demonstrate otherwise, with a multi-centred, larger population size.

Although abuse potential within the BZD class has not been systematically studied, prolonged use of benzodiazepines with greater lipophilicity and a shorter half-life appear to possess greater abuse potential.^17^

Whilst this study showed a duration of > 24 months of use being just marginally significant, this finding correlates with findings of studies indicating a link between prolonged use and physical or psychological dependence.^17^ Whilst oxazepam has been considered an agent of low abuse potential,^18^ it does have a shorter half-life compared to clonazepam that poses as a high-potency benzodiazepine with a long half-life contributing to a ‘safer’ profile.^19^ This may account for the marginally significant difference found between the two agents in this study.

When considering that the SDS has shown utility in measuring dependence with various other drugs,^15,16^ this research indicates that this screening tool, with adjustment to terminology, can be considered as a precise and useful screening test for BUD.

In comparing the results of the study to those conducted by De Las Cuevas et al.^13^ and Tsai et al.,^14^ our cut-off score was established at 6 compared to 6–7 found in these studies. The sensitivity and specificity in this study were lower than that of the study by De Las Cuevas et al.^13^ and greater than that by Tsai et al.^14^ The AUC depicted in this research was analogous with results found by De Las Cuevas et al.^13^

Factors contributing to the difference in results may include tools used for diagnosis with this study focusing on BUD *DSM 5* criteria and the previous studies addressing benzodiazepine dependence. Additionally, characteristics of the population sample such as comorbid psychiatric diagnoses and sociocultural factors could have impacted on findings. On further analysis of our data, it was noted that 5/7 false negatives for a BUD diagnosis were individuals with a duration of usage greater than 24 months. Inferences from a small sample pose a challenge and a potential remedy would be to extend the study to further clinics to increase the sample size and enable more detailed analysis across variables with possible emphasis describing and analysing average scores of the SDS in relation to the various demographic variables.

## Strengths and limitations

This study may be limited by the sample studied representing patients attending at an outpatient mental health service and therefore may not be easily extrapolated to other populations of benzodiazepine users. To enhance the generalisability, a multi-centred study with a larger population size would be warranted.

Identification of participants and data collection were based on record keeping by both nursing and psychiatry doctors, and bias may have been introduced on review of the files in terms of a possible pre-existing BUD diagnosis. Additionally, responses may be affected by patient’s truthfulness in fear of adjustment of current treatment regardless of reassurance.

The SDS was also only available in an English format, and this may have been challenging for participants whose first language was not English. Moreover, results may be impacted on by perceived stigma associated with an additional diagnosis of a possible SUD.

The diagnosis was dependent on a brief interview with the patient as opposed to a validated, standardised diagnostic instrument; however, the *DSM 5* checklist was utilised with every patient to account for this. This could be addressed in future studies by repeated interviews with validated instruments, standardised questions and various collateral sources.

Despite these limitations, the study had some strengths. It is, to the best of our knowledge, the first of its nature in the South African setting. It created an opportunity to share information in this population of benzodiazepine users and the treating team with emphasis on highlighting risks of BUD. Additionally, it presents an opportunity to assess and refer on BUD patients using an easily administered, short questionnaire conducted by any healthcare professional in a resource limited setting.

## Conclusion and recommendations

Early identification and appropriate assessment of BUD will aid in initiating referral of the patient and management of the condition. Although screening tools are not to replace the clinical assessment and validated diagnostic tools, a quick and easily administered screening questionnaire would assist greatly in referral, especially in resource-constrained settings.

The SDS illustrated a high diagnostic utility with a cut-off point of 6 in identifying problematic BZD users and can therefore be considered as a valid, brief self-reported questionnaire for the assessment of BUD amongst regular BZD users with mental illness.
